# Presence of unique glyoxalase III proteins in plants indicates the existence of shorter route for methylglyoxal detoxification

**DOI:** 10.1038/srep18358

**Published:** 2016-01-06

**Authors:** Ajit Ghosh, Hemant R Kushwaha, Mohammad R Hasan, Ashwani Pareek, Sudhir K Sopory, Sneh L Singla-Pareek

**Affiliations:** 1 Plant Molecular Biology Group, International Centre for Genetic Engineering and Biotechnology, Aruna Asaf Ali Marg, New Delhi-110067, India; 2 Stress Physiology and Molecular Biology Laboratory, School of Life Sciences, Jawaharlal Nehru University, New Delhi-110067, India; 3 Present address: Department of Biochemistry and Molecular Biology, Shahjalal University of Science and Technology, Sylhet-3114, Bangladesh

## Abstract

Glyoxalase pathway, comprising glyoxalase I (GLY I) and glyoxalase II (GLY II) enzymes, is the major pathway for detoxification of methylglyoxal (MG) into D-lactate involving reduced glutathione (GSH). However, in bacteria, glyoxalase III (GLY III) with DJ-1/PfpI domain(s) can do the same conversion in a single step without GSH. Our investigations for the presence of DJ-1/PfpI domain containing proteins in plants have indicated the existence of GLY III-like proteins in monocots, dicots, lycopods, gymnosperm and bryophytes. A deeper *in silico* analysis of rice genome identified twelve DJ-1 proteins encoded by six genes. Detailed analysis has been carried out including their chromosomal distribution, genomic architecture and localization. Transcript profiling under multiple stress conditions indicated strong induction of *OsDJ-1* in response to exogenous MG. A member of OsDJ-1 family, *OsDJ-1C*, showed high constitutive expression at all developmental stages and tissues of rice. MG depletion study complemented by simultaneous formation of D-lactate proved OsDJ-1C to be a GLY III enzyme that converts MG directly into D-lactate in a GSH-independent manner. Site directed mutagenesis of Cys-119 to Alanine significantly reduces its GLY III activity indicating towards the existence of functional GLY III enzyme in rice—a shorter route for MG detoxification.

Discovered more than hundred years back in rabbit and dog tissues, glyoxalase pathway is one of the most ubiquitous and evolutionary well conserved pathway^1^,^2^. The pathway essentially consists of two enzymes, glyoxalase I (GLY I) and glyoxalase II (GLY II). GLY I converts a highly cytotoxic metabolite methylglyoxal (MG) into S-lactoylglutathione (SLG) with the help of reduced glutathione (GSH)^3^.The SLG is further hydrolyzed into D-lactate by GLY II and GSH is recycled back into the system (Fig. 1). Higher concentrations of SLG can inhibit DNA synthesis, hence is considered toxic to the living system^4^.

Apart from MG, glyoxalases can act on various other α-oxoaldehydes produced in the living system, such as glyoxal and hydroxy-pyruvaldehyde^5^. These carbonyl compounds are very reactive in nature and accumulate during various diseases in animals and stress conditions in plants^6^,^7^. They are mainly generated as a by-product of glycolysis pathway or glucose degradation^8^. They can also be generated through amino acid metabolism, fatty acid catabolism and lipid peroxidation^8^. Due to highly electrophilic nature, they form adducts with the nucleophilic centres of cellular macromolecules such as DNA, RNA and proteins^9^. They can modify the arginine, lysine, cysteine residues of protein and adenine, guanine bases of nucleic acids; and thus forms various Advanced Glycation End products (AGEs)^8^. Accumulation of AGEs is directly correlated with apoptosis of cells and thus involved in the disease progression in human^10^. To avoid these undesired cellular destructions, organisms have evolved the glutathione-dependent glyoxalase pathway^3^ and NAD(P)H-dependent Aldo-Keto reductase pathway^11^.

Although glyoxalase pathway is considered as an omnipresent system in all living organisms, some organisms that lack GLY I or GLY II, have been reported. For example, enteric protozoans *Entamoeba histolytica*, *Giardia lamblia* and *Trypanosoma brucei* do not have any GLY I gene^12^ and some mammals (such as horse) do not have any GLY II gene^13^. Along with this well conserved pathway, a novel type of glyoxalase III (GLY III) activity has been observed in *E. coli* total cell lysates for a long time^14^ that could irreversibly covert MG into D-lactate in a single step without the help of any cofactor (Fig. 1). Earlier, it has also been shown that a known heat inducible chaperone Hsp 31 showed GLY III-like activity in *E. coli*^15^. Although GLY III exhibits higher activity as compared to GLY I/II system *in vitro*, it shows less activity *in vivo* of *E. coli*^16^. Moreover, it has been reported that this enzyme has lower efficiency (~1000 fold) in comparison to GLY I and GLY II^17^,^18^. Nonetheless, unlike the conventional glyoxalase (GLY I/II) system, GLY III is not dependent on any metal ions for its optimal activity^15^. Further analysis of *E. coli* GLY III protein revealed that this is a member of DJ-1/PfpI super family and possess a conserved catalytic triad his-cys-glu in its active site^15^.

DJ-1/PfpI domain containing proteins have been found to exhibit other physiological roles, such as regulation of mitochondrial function^19^, regulation of transcription^20^, molecular chaperone^15^, stimulation of anti-oxidant enzymes^21^ and protease^22^ in various organisms. DJ-1 proteins play important role towards protection against methylglyoxal induced cell death in human, mouse and *C. albicans* cells^18^. Moreover, mutation in human DJ-1 protein leads to parkinson’s disease^23^ and various forms of cancers^24^. Besides their role in cyto-protection, they might be involved in other cellular process and thus, essential for the viability of various organisms, including human, rat, *C. albicans*, zebrafish and fly^25^,^26^,^27^,^28^. In case of plants, loss of AtDJ-1a function causes cell death, whereas overexpression leads to increased oxidative stress tolerance^21^. It has been also observed that expression of AtDJ-1a is induced by various stresses and interacts with several stress inducible proteins such as superoxide dismutase and glutathione peroxidase^21^. However, the exact role and physiological relevance of GLY III along with well conserved glutathione-dependent glyoxalase pathway, is not clear yet. Limited information about its presence and role under control and stress conditions, suggests it may be functionally non-redundant^29^.

In the present study, we have performed analysis of DJ-1/PfpI domain containing proteins across plant kingdom in order to explore the existence of this unique alternate and short route of the detoxification of deleterious metabolite-MG. In order to gain insights into the functional relevance of DJ-1 proteins in rice, we have analyzed expression profiling of these genes at different developmental stages, various tissues and multiple stress conditions. Moreover, presence of functional GLY III activity for one of the OsDJ-1 proteins, OsDJ-1C, has been confirmed by enzyme kinetics and site-directed mutagenesis. It has been shown that OsDJ-1C could convert MG into D-lactate in a single step driven glutathione-independent reaction. The investigations performed on the DJ-1 members will assist in elaborating the understanding about the evolution of this unique glyoxalase pathway.

## Results

### Identification of DJ-I/PfpI domain containing proteins in plants

The present study outlines the presence of DJ-1/PfpI domain containing proteins in plants at various taxonomic levels. The sequences of DJ-1 proteins were searched against the swiss-prot database using functional *Arabidopsis* GLY III, *AtDJ-1d* (AT3g02720). All the sequences obtained were cross-checked for the presence of DJ-1/PfpI domain (PF01965.19). A total of 217 sequences from various plant taxa were used to perform sequence and phylogenetic analysis. Given below is the brief description of DJ-1/PfpI domain containing proteins in monocots, dicots and other species.

#### DJ-1/PfpI domain containing proteins in monocots.

Out of 217 DJ-1 proteins considered, ninety nine were found to be present in monocots (Table 1). The monocot plant species used for analyzing the DJ-1 proteins includes eight species of *Oryza* such as *Oryza punctata*, *Oryza officinalis*, *Oryza rufipogon*, *Oryza minuta*, *Oryza sativa* subsp. *indica*, *Oryza brachyantha*, *Oryza glaberrima* and *Oryza sativa* subsp. *japonica*. The maximum number (twelve) of DJ-1 proteins were found to be present in *Oryza sativa* subsp. *japonica* while *Oryza glaberrima* has six, *Oryza sativa* subsp. *indica* and *Oryza brachyantha* have five, *Oryza minuta* has two and minimum member present in *Oryza punctata*, *Oryza officinalis* and *Oryza rufipogon*. Two *Triticum* species; *Triticum urartu* and *Triticum aestivum* were found to have three and thirteen members of the DJ-1 proteins, respectively. Among the monocots, maximum number i.e. twenty-one DJ-1 proteins were observed in *Hordeum vulgare* var. *distichum*. Among others, *Zea mays* has eleven, *Setaria italica* and *Sorghum bicolor* have five, *Musa acuminata* has four, *Brachypodium distachyon* has three and *Aegilops tauschii* has only one DJ-1/PfpI domain containing proteins (Table 1).

### DJ-1/PfpI domain containing proteins in Dicots

Out of 217 DJ-1 protein members, one hundred and seven proteins were found to be present in dicots (Table 1). Apart from the model plant *Arabidopsis thaliana*, other species of *Arabidopsis* such as *Arabidopsis lyrata* and *Arabidopsis halleri* were also considered in the analysis. The maximum number i.e. ten members of DJ-1 proteins were observed in *Arabidopsis thaliana* and *Glycine max*. Further, multiple DJ-1 protein members were identified in *Brassica rapa*, *Populus trichocarpa*, *Arabidopsis lyrata*, *Phaseolus vulgaris*, *Citrus clementina*, *Ricinus communis*, *Capsella rubella*, *Thellungiella salsuginea*, *Medicago truncatula*, *Vitis vinifera*, *Solanum tuberosum*, *Solanum lycopersicum*, *Prunus persica*, *Genlisea aurea*, *Amborella trichopoda* (Table 1). Only one DJ-1 protein member was identified in *Thellungiella halophila*, *Arabidopsis halleri*, *Capsicum chinense, Lotus japonicus* and *Gossypium hirsutum*.

### DJ-1/PfpI domain containing proteins in other species

A total of eleven DJ-1 proteins were found to be present in other plant species apart from monocots and dicots such as Bryophyta (*Physcomitrella patens*), Gymnosperm (*Picea sitchensis*) and Lycopod (*Selaginella moellendorffii*). Among them, *Selaginella moellendorffii* has five, *Picea sitchensis* has four and *Physcomitrella patens* has two DJ-1/PfpI domain containing proteins (Table 1). The number of DJ-1/PfpI domain containing proteins represented in Table 1 for various species may not represent the actual number in case of unsequenced genomes.

### Sequence and Phylogenetic analysis of DJ-1/PfpI proteins

**Figure 2:**
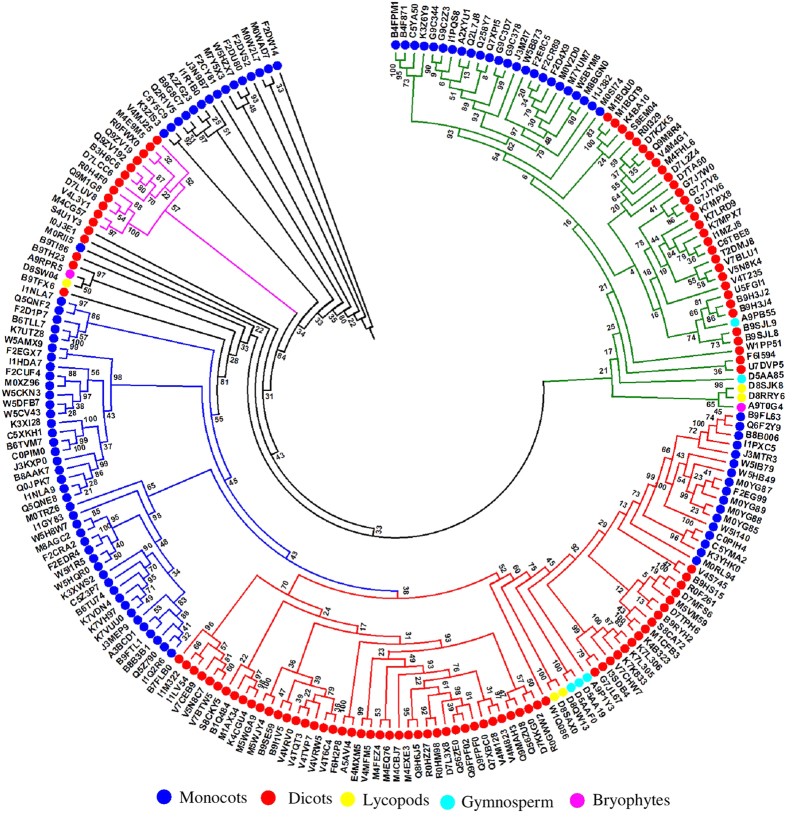
Phylogenetic analysis of DJ-1/PfpI domain containing proteins in plants. Phylogenetic tree obtained from DJ-1/PfpI domain containing 217 putative protein sequences (Table 1) from various species. Plants from five different major kingdoms, such as dicots, monocots, lycopods, gymnosperms, and bryophytes were used in the study and marked with different color in the tree.

The maximum likelihood (ML) tree plotted using all the 217 sequences showed four major clades. Interestingly, clade I (marked as green sub-tree) (Fig. 2), showed the presence of DJ-1 proteins from both monocots and dicots marking the highly conserved level in the protein sequences. These sequences in the clade I were found to share 43% to 79% identity among themselves. Clade I could be further divided into two sub-trees that clearly showed the segregation of DJ-1 proteins among monocots and dicots. Among the monocots, the sequence identity of DJ-1 members was 76% to 100% while in dicots the identity varied from 58% to 76%. The DJ-1 proteins from bryophyte, gymnosperm and lycopod showed more sequence identity with those of dicots (21% to 30%) and resided on the sub-branch of dicot species.

Similar to clade I, clade II also showed the clear segregation (branches marked in red) of DJ-1 proteins from both monocots and dicots (Fig. 2). In the clade II, the DJ-1 members from monocots showed identity in the range of 42% to 86% while those from dicots showed 46% to 76% identity among themselves. Similar to clade I, some DJ-1 members from gymnosperm and lycopod species had low identity (26% to 35%) with those from the dicots and are present in distinct separate sub-clade.

The clade III (branches marked in blue) showed DJ-1 proteins from monocots only and they share sequence identity of 46% to 88%. The members from monocots are quite conserved and formed separate group or sub-group among themselves in phylogenetic tree (Fig. 2). Although the clade II and clade III can be merged into a big clade but these clades are kept separated for easy analysis. The study suggested that the DJ-1 proteins from monocots and dicots have evolved distinctly.

### Rice genome harbours twelve DJ-1 homologs

**Figure 3:**
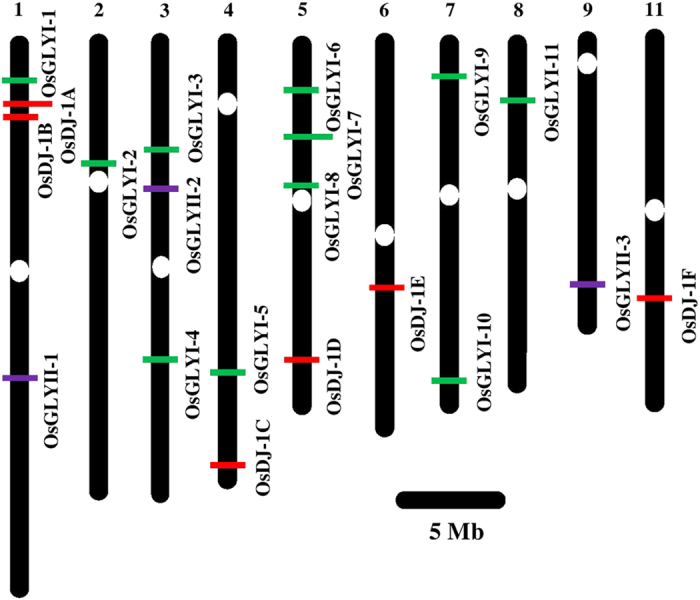
Chromosomal distribution of glyoxalase genes in rice. Unique glyoxalase III (*DJ-1*) genes have been marked along with the conventional glyoxalase I and glyoxalase II genes. Only the chromosomes having glyoxalase genes are shown here. Chromosome numbers are indicated at the top of the bar. *GLY I*, *GLY II* and *DJ-1* genes are marked with green, purple, and red colour boxes, respectively.

In order to have an overview from a highly important crop plant, *Oryza sativa* has been used for identification of members of DJ-1 protein family. We have identified six *DJ-1* genes in *Oryza sativa* subsp. *japonica* (Table 2). The members have been renamed for convenience as *OsDJ-1A* (LOC_Os01g11860), *OsDJ-1B* (LOC_Os01g11880), *OsDJ-1C* (LOC_Os04g57590), *OsDJ-1D* (LOC_Os05g44330), *OsDJ-1E* (LOC_Os06g34040) and *OsDJ-1F* (LOC_Os11g37920). All these *DJ-1* genes of rice were found to be localized on five chromosomes namely I, IV, V, VI, and XI (Fig. 3). Two genes, *OsDJ-1A* and *OsDJ-1B* were found be present on chromosome I and one each on chromosomes IV, V, VI and XI. In comparison with the chromosomal position of the conventional glyoxalase pathway genes (*GLY I* and *GLY II*), it was observed that rice chromosome I contain genes for *GLYI-1*, *GLYII-1*, and two GLY III-like *OsDJ-1A* and *OsDJ-1B* genes. None of the other chromosomes bears all the three genes.

In rice, six genes encoded for twelve OsDJ-1 proteins, indicating the involvement of alternate splicing. Only two of the members; *OsDJ-1C* and *OsDJ-1D* showed alternative splicing, and encodes for three and five proteins, respectively. These alternative splice variants have been raised due to the loss or gain of exons or introns, such as *OsDJ-1C*.1 losses one exon to generate *OsDJ-1C*.2, and loss of another exon make the second variant *OsDJ-1C*.3 (Figure S1). Similarly, *OsDJ-1D*.1 showed a successive one exon deletion and one intron retention to generate *OsDJ-1D*.2, two exons deletion to make *OsDJ-1D*.3 and four exons deletion to form *OsDJ-1D*.4 variant from its backbone coding sequence. All twelve OsDJ-1 proteins have an average molecular weight of ~ 40 kDa, except OsDJ-1D.4 (molecular weight of 25 kDa). Most of the proteins showed predicted acidic isoelectric point (pI) value, whereas OsDJ-1D.1, OsDJ-1D.2 and OsDJ-1E showed a basic pI value (Table 2). This indicates the presence of both positively and negatively charged DJ-1 proteins at the normal physiological pH of rice (7.4)^30^. Most of the OsDJ-1 proteins are found to be localized in cytosol and chloroplast, while OsDJ-1D.1 and OsDJ-1D.5 are predicted to be localized in mitochondria (Table 2). Chloroplast localization signal for OsDJ-1B, OsDJ-1D and OsDJ-1E was further confirmed by ChloroP (http://www.cbs.dtu.dk/services/ChloroP/)^31^.

### Sequence analysis of OsDJ-1/GLYIII-like proteins

**Figure 4:**
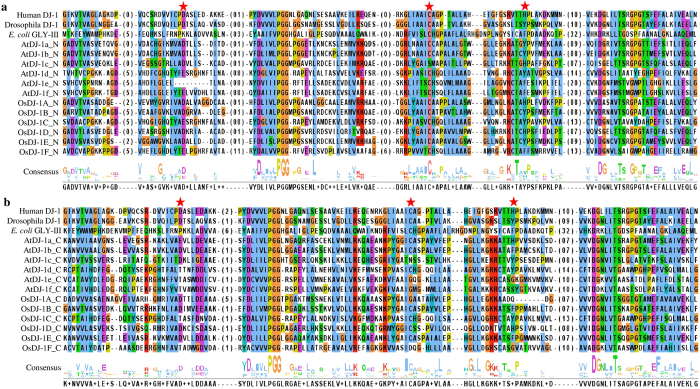
Sequence alignment of DJ-1/PfpI domain of OsDJ-1 proteins, compared with other DJ-1 homologs. All rice and *Arabidopsis* DJ-1 proteins have two DJ-1 domains and thus analyzed separately. (**a**) N-terminal domain and (**b**) C-terminal domain of all rice and *Arabidopsis* DJ-1 proteins were analyzed with three well characterized DJ-1 proteins from human (PDB ID: 1PDV), *Drosophila* (PDB ID: 4E08) and *E. coli* (PDB ID: 1N57). Residues directly involved in the catalysis (marked with red stars) were identified based on human and *Drosophila* DJ-1 protein structures.

All the rice and *Arabidopsis* DJ-1/GLY III-like proteins were found to contain two DJ-1/PfpI domains (Figure S2). However, GLY III proteins from *E. coli*, *Drosophila* and human were found to contain only single DJ-1/PfpI domain. The average size of the DJ-1/PfpI domain remains same in all the proteins (around 140 to 150 amino acids), except *E. coli* in which the domain size is 170 amino acids. Both the domains of OsDJ-1 proteins are connected by a linker of 25–70 amino acids, and have additional N-terminal peptides of 30–90 amino acids and C-terminal peptides of 5–20 amino acids. Both N and C-terminal DJ-1/PfpI domains of all rice and *Arabidopsis* DJ-1 proteins were individually compared with that of human, *Drosophila* and *E. coli* DJ-1/GLY III-like proteins (Fig. 4). Both human and *Drosophila* DJ-1 proteins contain a catalytic triad of aspartate/glutamate, cysteine and tyrosine/histidine residues (Fig. 4, marked with red stars). Among these, cysteine residue was found to be the most important and directly involved in the catalysis^32^. The N-terminal DJ-1 domain of all plant DJ-1 proteins (*Arabidopsis* and rice) showed the presence of this conserved cysteine residues, except AtDJ-1c (Fig. 4a). However, C-terminal domain showed very low conservation of this important residue where five of them AtDJ-1c, AtDJ-1e, AtDJ-1f, OsDJ-1A, and OsDJ-1F didn’t possess this residue (Fig. 4b). Absence of this important residue in both of its DJ-1 domain might be the probable reason for the complete absence of GLY III activity for AtDJ-1c^33^. Moreover, AtDJ-1e, AtDJ-1f and OsDJ-1F showed the absence of evolutionary conserved histidine/tyrosine residue that may have also led to the partial loss of GLY III activity in AtDJ-1e and AtDJ-1f.

### Developmental and tissue specific regulation of *OsDJ-1* genes in rice

**Figure 5:**
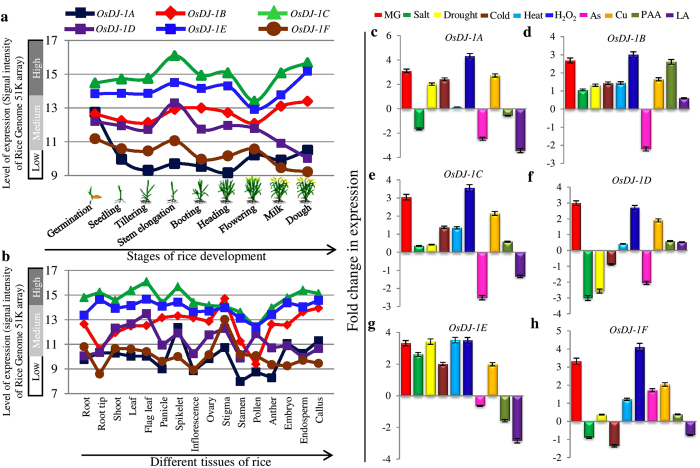
Expression profiling of *OsDJ-1* genes under different developmental stages, various tissues and multiple abiotic and biotic stresses. Expression was analyzed at nine distinct developmental stages (**a**) and sixteen tissues (**b**) of rice, as shown at the X-axis of the diagram. The mean signal intensity values from Affymetrix 51 K array were collected from genevestigator and plotted in the diagram. Color bar at the top of the figure represents the gene name. Expression of *OsDJ-1* genes was further analyzed under different stress conditions by qRT-PCR. Bar graphs (**c–h**) depict fold change in expression of *OsDJ-1* genes under different stress conditions – salt, cold, drought, heat, H_2_O_2,_ MG, As, Cu, PAA and LA. For expression analysis by qRT-PCR, 10 day old seedlings of IR64 variety (a moderately salt-sensitive cultivar) of rice were subjected to stress treatment for 8 hrs followed by RNA isolation, first strand cDNA synthesis and real-time PCR. Error bars showed standard deviation of three biological replicates.

In order to elaborate the role of OsDJ-1 proteins in various developmental processes of rice, we have checked the expression of *OsDJ-1* genes based on publicly available microarray database. Nine distinct rice developmental stages were selected for this study, such as germination, seedling, tillering, stem elongation, booting, heading, flowering, milk and dough stages. Expression analyses of *OsDJ-1* genes revealed that *OsDJ-1C*, followed by *OsDJ-1E*, showed higher constitutive expression in all the developmental stages analyzed (Fig. 5a). Other members such as *OsDJ-1B* and *OsDJ-1D* showed low to average expression at various developmental stages, whereas *OsDJ-1A* and *OsDJ-1F* showed considerably low expression at almost all the stages analyzed (Fig. 5a).

Further, expression profile of *OsDJ-1* genes was analyzed in different tissues such as root, root tip, shoot, leaf, flag leaf, panicle, spikelet, inflorescence, ovary, stigma, stamen, pollen, anther, embryo, endosperm and callus. Similar to developmental stages, medium to high transcript level was observed for *OsDJ-1C* and *OsDJ-1E* in various tissues analyzed, while a low to medium expression was observed for rest of the four members (Fig. 5b). Further, *OsDJ-1C* showed maximum transcript abundance among all *OsDJ-1* members in the tissues analyzed, except stigma where *OsDJ-1B* showed the maximum expression. Similar pattern of gene expression was observed by MPSS database analysis for *OsDJ-1* genes in various tissues (Figure S3). *OsDJ-1C* and *OsDJ-1E* showed higher transcript abundance in almost all the tissues analyzed, followed by *OsDJ-1B* and *OsDJ-1D*. Rest of the two members, *OsDJ-1A* and *OsDJ-1F* showed very low expression in all the analyzed tissues.

### Expression profiling of *OsDJ-1* genes in rice under various stress conditions

In order to understand the role of *DJ-1* genes in the stress physiology of rice, a qRT-PCR based expression analysis of *OsDJ-1* family members was performed under various stresses. For this purpose, a moderately salt-sensitive variety of rice IR64 was chosen. Relative transcript abundance of *OsDJ-1* genes was determined in response to various abiotic stresses such as salinity, drought, heat, cold, oxidative stress (H_2_O_2_), dicarbonyl stress (exogenous MG), heavy metal toxicity (As and Cu), and biotic stress inducing agents (PAA and LA) (Fig. 5c–h). All the members of *OsDJ-1* showed a significant three to four fold transcript up-regulation in response to dicarbonyl stress. It is well known that MG is a deleterious metabolite for plants and is detoxified to D-lactate through glyoxalase pathway. The significant increase in *OsDJ-1* gene expression in response to MG suggests that MG may acts as a direct substrate for OsDJ-1 proteins and thus induce the expression of gene through substrate inducible mechanism^34^.

In addition to dicarbonyl stress, *OsDJ-1* genes have been found to be up-regulated in response to most of the abiotic stresses. Among all the genes, *OsDJ-1E* showed two to four folds up-regulation in its transcript level under all the abiotic and oxidative stresses (Fig. 5g). Moreover, *OsDJ-1A*, *OsDJ-1B* and *OsDJ-1C* also showed increase in their transcript level in response to most of the abiotic as well as oxidative stresses (Fig. 5c–e). An up-regulation of *OsDJ-1* transcripts was observed in response to variation in temperature. Lowering the temperature (cold stress) led to up-regulation of all the members except *OsDJ-1D* and *OsDJ-1F*. Whereas, under high temperature conditions (heat stress), expression of all the *OsDJ-1* genes was found to be up-regulated, similar to oxidative stress condition (Fig. 5c–h). In case of heavy metal toxicity, different pattern of expression was observed in response to two different heavy metals, Cu and As. All the *OsDJ-1* genes showed significant up-regulation in response to Cu stress, while in response to As stress, a sharp down-regulation was observed for all the members, except *OsDJ-1F* which showed enhanced expression. In response to biotic stress inducing agents (fungal elicitors), 4-hydroxyphenylacetic acid (PAA) and linolenic acid (LA), *OsDJ-1A* and *OsDJ-1E* showed down-regulation while *OsDJ-1B* and *OsDJ-1D* showed up-regulation (Fig. 5c,d,f,g). On the other hand, *OsDJ-1C* and *OsDJ-1F* showed up-regulation in response to PAA but down-regulated under LA treatment (Fig. 5e,h).

### Presence of putative *cis*-acting regulatory elements in the promoters

**Figure 6:**
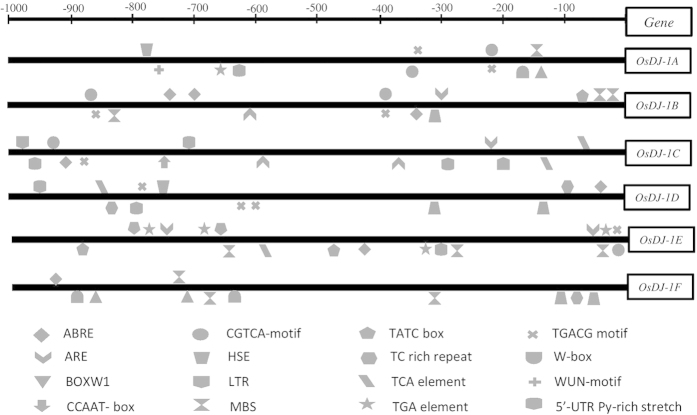
Promoter analysis of *OsDJ-1* genes. 1kb upstream region from transcription start site of *OsDJ-1* genes was selected and analyzed *in silico* for the presence of *cis*-acting regulatory elements. Different elements were represented as different artworks and indicated either above or below the line to represent the forward and reverse strands, respectively.

In order to investigate the reason behind the altered expression of *OsDJ-1* genes in response to various stresses, we have analyzed the 1 kb upstream region of the genes ‘*in silico*’ to identify the putative *cis*-acting regulatory elements. Putative promoter sequences (1 kb upstream of the transcription start site) for each of the OsDJ-1 family members were retrieved from the rice TIGR database^35^ and analyzed for the presence of *cis*-acting elements using PlantCARE database^36^ and PlantPAN^37^. This analysis revealed the presence of several known stress-related *cis*-acting elements such as abscisic acid responsive element (ABRE), anaerobic response element (ARE), fungal elicitor responsive element (BOX-W1), MYBHv1 binding site (CCAAT box), Methyl jasmonate-responsive element (CGTCA box and TGACG motif), heat shock element (HSE), low temperature responsive (LTR), MYB-binding site (MBS), gibberellin-responsive element (TATC-box), defence and stress response (TC-rich repeats), salicylic acid response element (TCA element), auxin responsive element (TGA element), W box, wound responsive element (WUN-motif) and transcription inducer (5′-UTR Py-rich stretch) (Fig. 6). All these motifs play very important role in the plant development and regulate various stress responses^38^,^39^ and predicted to be present in the promoter of various stress regulated rice gene families^40^,^41^. These motifs have been distributed randomly in the selected promoter region of *OsDJ-1* genes with minimum number in *OsDJ-1F* (seven elements). Maximum number of regulatory elements were found to be present in the promoter of *OsDJ-1C* and *OsDJ-1E* (nine elements), which showed the highest level of expression at various developmental stages and different tissues (Fig. 6) and up-regulation in response to abiotic stresses (Fig. 5c–h) respectively. The direct correlation between the gene expression and the presence of *cis*-acting regulatory elements needs to be analyzed further and confirmed experimentally.

### OsDJ-1C functions as GLY III enzyme

**Figure 7:**
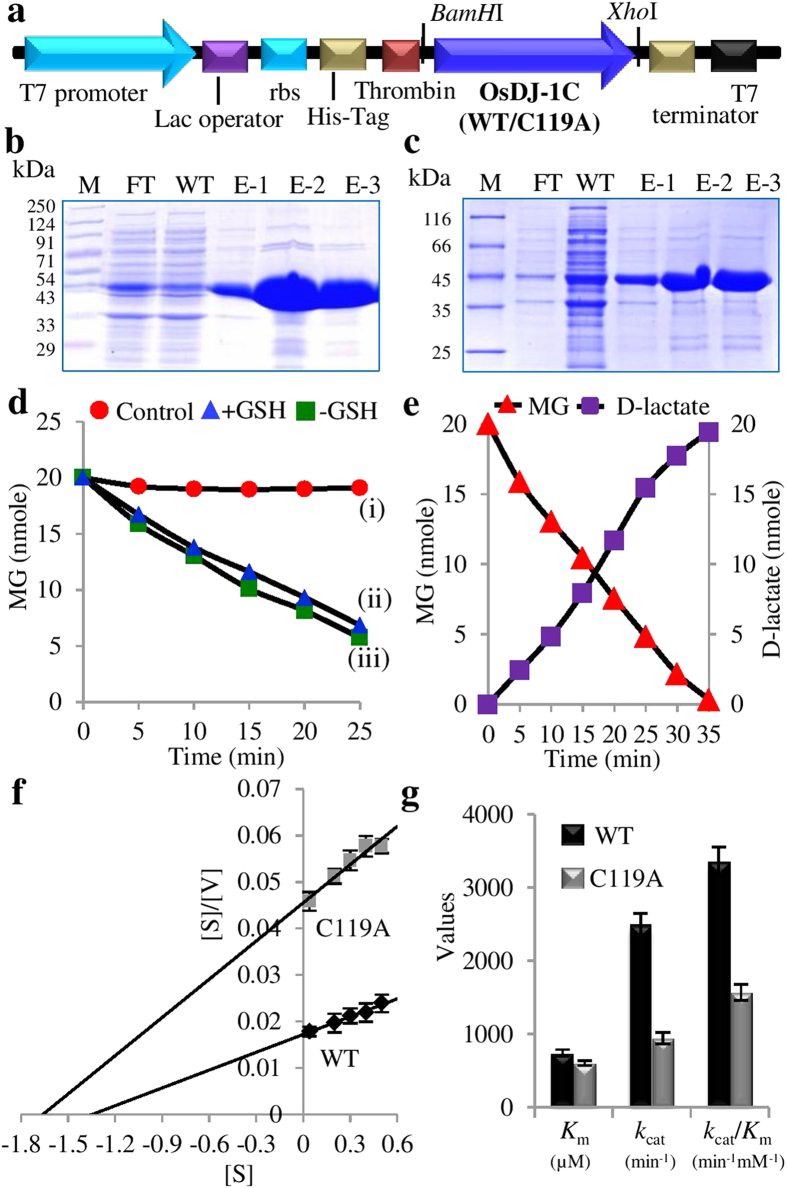
GLY III activity of recombinant OsDJ-1C protein and its enzyme kinetics. (**a**) Schematic diagram of pET28aOsDJ-1C construct used for expression and purification of His-tagged recombinant protein in *E. coli* BL21 (DE3) cells, where rbs: ribosome binding site, His-tag: histidine tag. Coomassie brilliant blue (CBB) stained PAGE showing Ni-NTA affinity purified recombinant wild-type OsDJ-1C protein (**b**) and C119A mutein OsDJ-1C protein (**c**); FT: flow through, WT: wash through (20 mM imidazole), E1-3: (elution fractions in 100 mM imidazole). (**d**) Depletion in MG content has been measured in different assay mixtures for 25 min, such as (**i**) without OsDJ-1C protein (red circle), (ii) with OsDJ-1C protein in the presence of GSH (blue triangle) and (iii) with OsDJ-1C protein in the absence of GSH (green square), indicating OsDJ-1C acts as GLY III enzyme in GSH-independent manner. (**e**) MG (substrate) utilization (red triangle) by OsDJ-1C enzyme with simultaneous generation of its product D-lactate (purple square) over a period of time (35 min). (**f**) Specific GLY III activity for OsDJ-1C protein (WT and its mutein C119A) was determined using various concentrations of MG as the substrate (0.04, 0.2, 0.3, 0.4 and 0.5 mM) and the kinetics was calculated using Hanes-Woolf plot. (**g**) Bar graph showing substrate specificity (*K*_m_), catalytic constant (*k*_cat_) and specificity constant (*k*_cat_/*K*_m_) of WT OsDJ-1C enzyme and its mutein C119A indicating significant difference in their enzyme kinetics. Experiments were repeated thrice with three replicates each time.

To check the GLY III activity of OsDJ-1 proteins, we have tested the enzyme activity of one of the representative member, OsDJ-1C. For this, *OsDJ-1C* gene has been cloned into a bacterial expression vector pET28a (Fig. 7a) and recombinant His-tagged protein was purified by routine Ni-NTA based affinity chromatography. Purified protein was found to be highly pure when examined on a 12% SDS polyacrylamide gel (Fig. 7b) and showed the expected prominent band of ~42 kDa. GLY III activity of this protein has been assayed using 20 nmole MG in 20 mM phosphate buffer by making three combinations of assay mixture (Fig. 7d). In first assay, only MG was added without protein (as control reaction), in second, MG was added along with the OsDJ-1C protein and in the third, MG was added along with the OsDJ-1C protein and GSH (+GSH). For all the three assay mixtures, the absorbance at 540 nm was measured at every 5 min interval till 25 min of reaction. No significant change in absorbance was observed in the control reaction mixture (Fig. 7di) indicating that MG is stable in the assay conditions and is not depleted in the absence of OsDJ-1C protein. A significant reduction in the MG content was noted when OsDJ-1C protein was added to the reaction mixture (Fig. 7diii) thereby indicating that OsDJ-1C protein acts as the GLY III enzyme that utilizes MG as the substrate. Since the glyoxalase enzymes require GSH as the cofactor, to find out the dependence of this GLY III enzyme on GSH, the same was added to the reaction mixture (Fig. 7dii). An almost similar decrease in MG concentration was observed in both –GSH and + GSH sets indicating that this GLY III enzyme could detoxify MG in a GSH-independent manner.

To further confirm the GLY III activity of recombinant OsDJ-1C protein, MG depletion was complemented by an assay based on the formation of D-lactate. For this, both utilization of substrate (MG) and generation of product (D-lactate) were measured in the same reaction mixture and a reverse correlation has been observed between them (Fig. 7e). This observation functionally validates that OsDJ-1C protein possess GLY III enzyme activity. The rate of MG utilization was observed to be exactly similar to that of D-lactate generation. This indicates a 1:1 stoichiometry of GLY III enzyme catalysis between substrate utilization and product generation (Fig. 7e). To calculate enzyme constants, initial velocities were measured by varying substrate (MG) concentration from 0.04 mM to 0.5 mM. Different enzyme kinetic constants of OsDJ-1C were calculated by plotting the initial velocity against substrate concentration in Hanes-Woolf plot (Fig. 7f). Values for *K*_m_ and *V*_max_ of OsDJ-1C were deduced from the plot as 0.74 mM, and 58 μmol min^−1^mg^−1^, respectively. From these data, *k*_cat_ of the protein was found to be 2500 min^−1^ and the corresponding *k*_cat_/*K*_m_ is 3.36 × 10^6^ min^-1^M^−1^ (Table 3 and Table S1).

### GLY III enzyme activity of OsDJ-1C is affected by mutation in its active site

A high level of sequence similarity was observed between the DJ-1 proteins from various species (Fig. 4). It has been found in various previous studies that one of the cysteine residues play important role in the catalysis of GLY III enzyme^15^,^33^,^42^. OsDJ-1C was also found to possess a conserved cysteine residue at C119 in its N-terminal domain (Fig. 4). To check whether this cysteine is involved in the catalysis of GLY III, C119 was altered to a neutral residue, alanine through site-directed mutagenesis. Both, the C119A mutein, as well as the wild type (WT) OsDJ-1C proteins were expressed in *E. coli* and purified (Fig. 7b,c). These recombinant proteins were assayed for the GLY III enzyme activity. Different enzyme kinetics parameters of C119A were calculated from Hanes-Woolf plot (Fig. 7f) and compared with those of WT OsDJ-1C protein (Fig. 7g and Table S1). As in Fig. 7g, both WT and C119A showed almost similar substrate specificity (*K*_m_). However, C119A mutein showed significant reduction (62%) in its catalytic constant (*k*_cat_) and (53%) in its catalytic efficiency (*k*_cat_/*K*_m_) as compared to the WT OsDJ-1C protein. This indicates that C119 is an active catalytic residue of OsDJ-1C protein thereby reconfirming this protein to be functional GLY III enzyme. Comparison of various kinetic parameters of OsDJ-1C with GLY III enzymes reported from other organisms (Table 3) showed significantly higher catalytic efficiency of OsDJ-1C in terms of its *k*_*cat*_ and *k*_cat_/*K*_m_ values except one of the *Arabidopsis* DJ-1 enzyme, AtDJ-1d (Table 3). Based on these catalytic parameters, OsDJ-1C is found to be a highly efficient enzyme as compared to other characterized DJ-1 proteins from different organisms (Table 3).

## Discussion

Understanding of the glyoxalase pathway can lead to better insights towards the cellular detoxification mechanisms of plants. The accumulation of reactive α-oxoaldehydes is highly deleterious to cell, as they react with cellular macromolecules such as nucleic acids and proteins^9^. Glyoxalase pathway and Aldo-keto reductase system are the key components to detoxify them using reduced glutathione and NAD(P)H as cofactors. The detoxification of α-oxoaldehydes is affected during various stress conditions due to the reduced levels of cofactors. Novel DJ-1/PfpI domain containing protein from *E. coli* showed glyoxalase III-like activity by converting these oxoaldehydes into their non-toxic form without the help of any cofactor(s)^15^. Earlier, DJ-1/PfpI superfamily proteins were found to play important role in animal system under oxidative stress and mitochondrial dysfunction^18^. Current analysis focuses on the identification of DJ-1/PfpI domain containing proteins across various plant taxa, with detailed analysis of rice DJ-1 members.

The sequence analysis of DJ-1 members in various plant species reveals the existence of *DJ-1* genes in monocots, dicots, lycopods, gymnosperms and bryophytes (Fig. 2). More interestingly, DJ-1 protein members have been observed to have evolved distinctly in the two major plant taxa, dicots and monocots. Overall, the domain structure (single or two domains of DJ-1/PfpI) is conserved in both monocots and dicots. Large numbers of DJ-1 proteins were found to have two DJ-1/PfpI domains while only 27 out of 217 sequences analyzed in this study were found to have single domain structure (Table S2), indicating towards the preference of two domain containing proteins in plants. Plant genomes though said to have evolved dynamically but have been observed to maintain conserved universal domain architectures^43^. The phylogenetic tree further elaborated on the variations present in DJ-1 proteins in both monocots and dicots. The clades and the sub-clades of the phylogenetic tree show the distinctness of DJ-1 proteins in monocots and dicots. Analyses of variations present in the sequences suggest higher variation among the DJ-1 proteins in dicots. Further, multiple members of DJ-1 protein homologs were observed in both well annotated genomes of rice and *Arabidopsis*. Both rice and *Arabidopsis* have six *DJ-1* genes (Fig. 3 and S4) that encode for twelve (Table 2) and eleven proteins (Table S3), respectively.

Previous studies suggested that the expression of *DJ-1* genes could be modified by external factors. Upregulation of *AtDJ-1* a transcript was observed in response to various exogenous stress cues, including oxidative stress^21^. Similar up-regulation of *OsDJ-1* genes has been observed in rice under oxidative stress (Fig. 5c–h). Glyoxalase pathway keeps MG level in check which regulates the cellular glutathione homeostasis. This resulted in the survival of plants under various abiotic stress conditions^44^,^45^. Apart from abiotic stresses, both up-regulation of GLY I protein and increased level of MG have been observed in maize after fungal infection^46^. Members of DJ-1 gene family in rice showed varied response towards both abiotic stresses and biotic stress inducers (Fig. 5c–h) which indicates towards their role in more than one mechanism of keeping MG level in check or may be due to division of labour among the genes in a multi-gene family. Results presented here show the presence of *OsDJ-1* genes and their alteration of expression at various stages of rice development (Fig. 5a). This may be due to the detoxification mechanisms adopted by the plant which finally affects the overall growth and development of the plant. *In silico* interaction analysis of OsDJ-1 proteins revealed that OsDJ-1 proteins may interact with a total of five hundred three proteins of rice (Fig. S5). Further analysis of these interacting proteins indicated that they are mainly involved in metabolic process (228 proteins) followed by transportation (59 proteins). However, the exact role of DJ-1 proteins along with its interacting partners needs to be investigated further.

*In silico* analysis predicted the presence of several stress responsive *cis*-acting regulatory elements in the promoter region of *OsDJ-1* genes (Fig. 6). *Cis*-acting elements play important role in regulating the expression of genes under various stresses and developmental changes^47^. For example, we have observed the prediction of heat shock element (HSE) in the promoter of *DJ-1* genes that may lead to the up-regulation of *OsDJ-1* genes in response to heat stress (Fig. 5c–h). Besides that, DJ-1 proteins have been previously reported to act as a heat shock protein (hsp) in case of *E. coli*^15^. Three highly abiotic stress responsive elements such as ABRE, MBS and LTR were found to be predicted in the promoter of *OsDJ-1* genes that may provide up-regulation of the corresponding genes under abiotic stresses. Moreover, plant phytohormones have been reported to play important role in response towards various abiotic and biotic stresses. Among them, abscisic acid (ABA) was involved in various abiotic stresses, Jasmonic acid (JA) regulates plant response against pathogens and herbivores and salicylic acid (SA) has also a potential role in the biotic and abiotic stress response of plants. Interestingly, the promoter region of *OsDJ-1* genes also predicted to contain some hormone responsive elements such as CGTCA box, TGACG motif, TATC box, TCA element, TGA element, which might sense the various signalling response and regulate the expression of respective genes in response to stress as well as developmental cues. Further, the presence of BOXW1, W-box and WUN-motifs in the promoter region of *OsDJ-1* genes could be directly correlated with the alteration of *OsDJ-1* transcripts in response to fungal elicitor. This indicates the potential role *OsDJ-1* genes in the abiotic, as well as biotic stress response of plant.

Functional GLY III activity of DJ-1 proteins has been reported from various species such as *E. coli*^15^, human^18^, *Arabidopsis*^33^, *Candida albicans*^29^, and *Schizosaccharomyces pombe*^48^. Different types of functional activities have also been assigned to DJ-1 proteins, such as molecular chaperone, amino peptidase and proteases etc^22^,^49^. To confirm the GLY III activity of rice DJ-1 proteins, we have selected one of the highly expressive members, OsDJ-1C for further validation. Enzyme activity data showed that OsDJ-1C can utilize the deleterious metabolite-MG as the substrate to produce D-lactate in a glutathione independent manner (Fig. 7d,e). Site-directed mutagenesis of the conserved cysteine in the N-terminal domain of OsDJ-1C was found to affect its GLY III activity (Fig. 7f,g), thus reconfirming it be a functional enzyme. This validates the presence of functional GLY III enzyme in rice that can carry out MG detoxification following a shorter GSH-independent route in contrast to the conventional GSH-dependent two step glyoxalase I/II pathway. Although, the efficiency of OsGLY III enzyme has been found to be relatively low as compared to the conventional GLY I/II enzymes in terms of their enzyme kinetic parameters (Table S4), it was still higher than so far reported GLY III enzymes except AtDJ-1d (Table 3). Conventional GLY I/II enzymes could work efficiently at very low substrate concentration, whereas GLY III did this at relatively higher concentration of MG. Difference in the substrate specificity might play an important role in determining the mode of action of the two alternate pathways. This indicates, conventional GLY I proteins are the primary enzyme to detoxify MG under normal physiological conditions. Intracellular level of MG goes up during stress condition^44^, that may require GLY III along with conventional GLY I/II pathway to tackle the accumulation of excess MG. When we compared various enzyme constants of OsDJ-1C protein with other reported DJ-1 proteins, it showed significantly higher affinity (lower *K*_m_) to substrate (MG) and better enzyme catalytic efficiency (higher *k*_cat_) as compared to other reported GLY III enzymes, except AtDJ-1d (Table 3). Use of highly purified MG in the present study might be one of the reasons for the same. Moreover, catalytic efficiency (*k*_cat_/*K*_m_) of OsDJ-1C was found to be reduced by more than 50% without significant alteration in their substrate specificity (*K*_m_), when one of the conserved Cys residues (C119) has been mutated to Ala (Table S1). This confirms the clear involvement of C119 in the catalytic mechanism of GLY III, but not in the substrate binding.

Taken together, present study represents a detailed identification of DJ-1/PfpI domain containing proteins in complete plant kingdom. Wide spread presence of these proteins in all plant classes indicates the importance of DJ-1 proteins which provides an alternate, rapid and glutathione-independent route to keep cytotoxic MG level under control. Further, expression, enzyme kinetics and mutation studies highlighted the role of DJ-1 proteins as GLY III enzymes in rice along with the conventional glyoxalase pathway. The lead provided in this paper can thus assist in developing future strategy to study various other members of DJ-1 family in different plant species.

## Methods

### Homologous gene identification

Using a functional GLY III protein of *Arabidopsis*, AtDJ-1d (AT3g02720) as reference, a total of 273 DJ-1 sequences were obtained from various plant species using Uniprot Blast database (www.uniprot.org) with the default cut-off. The poorly aligned and algal DJ-1 sequences were discarded that lead to the total of 217 sequences from plants. Sequences were aligned using MUSCLE v3.8^50^ and viewed using JALVIEW program^51^.

### Gene tree construction

We used Maximum Likelihood (ML) method for phylogenetic reconstruction with 100 sets of data which were obtained using “seqboot” program of PHYLIP^52^. The ML tree was obtained using PHYML^53^ with 100 bootstrapping and default parameters. The final tree obtained was visualized and analyzed using program MEGA^54^.

### Nomenclature of *DJ-1* genes and sub-cellular localization of OsDJ-1 proteins

According to the pattern followed earlier^55^, we referred rice DJ-1 genes as *OsDJ-1*, where Os is for *Oryza sativa*, followed by DJ-1 and suffixed English letter (A to F). The alternative spliced forms were represented with the numeral following the gene name. Localization of OsDJ-1 proteins was predicted by CELLO v.2.5: sub cellular localization predictor (http://cello.life.nctu.edu.tw/)^56^ and cross checked using pSORT prediction software (http://wolfpsort.org/)^57^.

### Microarray-based expression profiling of *OsDJ-1* genes

The expression of *OsDJ-1* genes was analyzed using publicly available microarray data. To study the expression pattern at different developmental stages and various tissues, the normalized and curated signal intensities values on the 51 K array were retrieved using Genevestigator (https://www.genevestigator.com/gv/plant.jsp) as described previously^44^ and plotted in scatter diagram.

### Plant material, stress treatment and qRT-PCR

*Oryza sativa* L. cv IR64 seeds were surface sterilized with 0.25% Bavistin for 20 min and allowed to germinate in hydroponic system under control conditions in growth chamber (SANYO, North America Corporation) at 28 ± 2 °C temperature and 16 h/8 h photoperiod. Ten day old seedlings were shifted to Yoshida medium^58^ containing different stress inducing agents for 8 hrs. The following stress were imposed such as 200 mM NaCl was added for salinity stress; 0.1 mM H_2_O_2_ for oxidative stress, 0.01 mM MG for dicarbonyl stress, 30 mM sodium arsenate dibasic heptahydrate (Na_2_HAsO_4_.7H_2_O) or 0.01 mM CuSO_4_ for heavy metal toxicity, 10 mM 4-hydroxyphenylacetic acid (PAA) or 2 mM linolenic acid (LA) to mimic fungal infection. For drought stress, the seedlings were air-dried; for cold and heat stress, the seedlings were shifted to growth chamber at 4 °C and 42 °C respectively. Seedlings grown in normal Yoshida medium at optimal conditions were used as the experimental control. After 8 hrs of stress treatment, shoot tissues were harvested, and total RNA was isolated using TRIzol reagent (Life Technologies, USA) as per the manufacturer’s protocol. The quality and integrity of RNA was analyzed using denaturing agarose gel electrophoresis, and first strand cDNA was synthesized using RevertAid™ RNase H minus cDNA synthesis kit (Thermo Fisher Scientific Inc, USA). The qRT-PCR was performed as described previously^41^ using gene specific primers unique to each of the six members of *OsDJ-1* family along with *eEF-1*α as the endogenous control (Table S5). The specificity of the amplification was tested by dissociation curve analysis and the fold change in expression was calculated as done previously^41^. The experiment was repeated with three biological replicates and the mean fold change was plotted.

### *In silico* promoter analysis for the presence of putative *cis*-regulatory elements

To search for the presence of various *cis*-acting regulatory elements in promoter sequences of *OsDJ-1* genes, 1 kb upstream regions from transcription start site were retrieved from rice genome chromosomal co-ordinate database (http://rice.plantbiology.msu.edu/cgi-bin/gbrowse/rice/). The sequences were subsequently scanned through PlantCARE^36^ and PlantPAN^37^ databases for the prediction of stress related *cis*-acting regulatory elements.

### Cloning, expression and purification of OsDJ-1C protein

*OsDJ-1C* was amplified by PCR using total rice (cv. IR64) cDNA as template and gene specific primers (DJ-1C_For_*BamH*I: 5′CGGGATCCATGGCTCCCAAGAAGGTGCT3′ and DJ-1C_Rev_*Xho*I: 5′CCGCTCGAGTCAGAAGGAGACCTTGATGC3′). Amplified fragment was cloned between *BamH*I and *Xho*I site of pET28a vector, transformed into *E. coli* BL21 cells and confirmed by sequencing. Protein expression was induced with 0.1 mM of IPTG at 18^°^C for overnight and purified by Ni-NTA as described previously^44^.

### Site-directed mutagenesis of OsDJ-1C, expression and purification of the mutein

Mutagenesis of *OsDJ-1C* gene (Cysteine 119 residue to Alanine) was carried out using special 5′phosphorylated PAGE purified primers (forward 5′GCATCAGTTGCCCATGGACAGT3′ and reverse 5′AATAGGTTTCTTCGCATCAGAG 3′) and *OsDJ-1C*_pET28a plasmid as the template according to manufacturer’s protocol (Phusion site directed mutagenesis kit, Finnzymes, Finland). Mutation was confirmed by sequencing, followed by expression and purification of the mutein as described above.

### Purification of Methylglyoxal

Highly purified methylglyoxal was prepared by acid hydrolysis of methylglyoxal-1,1-dimethyl acetal (MGDA, Sigma-Aldrich, catalog no. 170216) in the presence of sulfuric acid (95–98%) followed by fractional distillation under reduced pressure^59^. In brief, MGDA was diluted in 2.5% aqueous sulfuric acid solution and allowed to heat in a boiling water bath for one hour. After that, the mixture was fractionally distilled and collected into three separate fractions under reduced pressure as described by Rabbani and Thornalley^60^. Highly purified MG (third fraction) was used as the MG source for all further biochemical experiments.

### Glyoxalase III activity of OsDJ1-C protein

GLY III activity was measured on the basis of MG depletion assay. For this, recombinant OsDJ-1C protein (2 μg) was incubated with MG (20 nmole) in 20 mM sodium phosphate buffer (pH 7.2) in a 1000 μl reaction mixture as described^15^. In brief, utilization of MG was measured at 42^0^C at fixed time points (0, 5, 10, 15, 20 and 25 min) in three different assay mixtures viz. (i) without OsDJ-1C protein as the experimental control, (ii) with OsDJ-1C protein and (iii) OsDJ-1C protein along with GSH (20 nmole). After fixed time points (as mentioned above), 30 μl of reaction mixture from each was immediately added to a freshly prepared reaction mixture of 1 ml. The reaction mixture contains 1.8 mM of 1,2-diaminobenzene (DAB), 0.5 M of perchloric acid, and 1 mM of sodium azide (NaN_3_)^44^. After addition of sample, the reaction mixture was incubated for 3 h and the absorbance was read at 336 nm of wavelength as described previously^44^. The absorbance values were interpolated from the MG standard curve prepared in the similar way to determine the MG concentration.

To further prove the GLY III activity of OsDJ-1C protein, MG depletion assay was complemented with the formation of D-lactate. For this, an assay for MG depletion with a simultaneous generation of D-lactate was carried out. A 1000 μl reaction mixture was prepared by adding the recombinant OsDJ-1C protein (2 μg) with MG (20 nmole) in 20 mM sodium phosphate buffer (pH 7.2) and an aliquot from this was used for the measurement of both MG and D-lactate at fixed time points (0, 5, 10, 15, 20, 25, 30 and 35 min). MG content was measured following the method described above. D-lactate was measured following the method described earlier^29^ with slight modifications. Briefly, 30 μl of reaction mixture from each time point was heat-inactivated immediately at 80 °C for 60s and allowed to cool on ice for 5 min. The inactivated cooled reaction mixture was added to 50 μl NAD^ + ^, 10 μl of D-lactate dehydrogenase and 20 mM sodium phosphate buffer (pH 7.6) in a reaction mixture of 1000 μl followed by incubation for atleast 10 min at room temperature or longer until the reaction reaches the endpoint (absorbance does not vary further). D-lactate dehydrogenase in the presence of NAD^ + ^will oxidize D-lactate (that was produced by GLY III activity) to pyruvate and NADH. The absorbance of NADH was taken at 340 nm and converted to a molar concentration using an extinction coefficient of NADH at 340 nm of 6300 [lxmol^−1^x cm^−1^].

### Enzyme kinetics of OsDJ1-C protein and its mutein C119A

Varying concentrations of MG (0.04 to 0.5 mM) in the presence of enzyme (as sample—either WT OsDJ1-C protein or its mutein C119A) and without enzyme (as control) were used to study the kinetics of GLY III enzyme (both WT and mutant version) by monitoring the utilization of MG for 10 min. MG content was measured by using the method as described above. Different kinetic constants such as *K*_m_, *V*_max_, *k*_cat_ and *k*_cat_/*K*_m_ were determined by analyzing the data through Hanes-Woolf plot as described previously^44^.

## Additional Information

**How to cite this article**: Ghosh, A. *et al.* Presence of unique glyoxalase III proteins in plants indicates the existence of shorter route for methylglyoxal detoxification. *Sci. Rep.* **6**, 18358; doi: 10.1038/srep18358 (2016).

## Supplementary Material

Supplementary Information

## Figures and Tables

**Figure 1:**
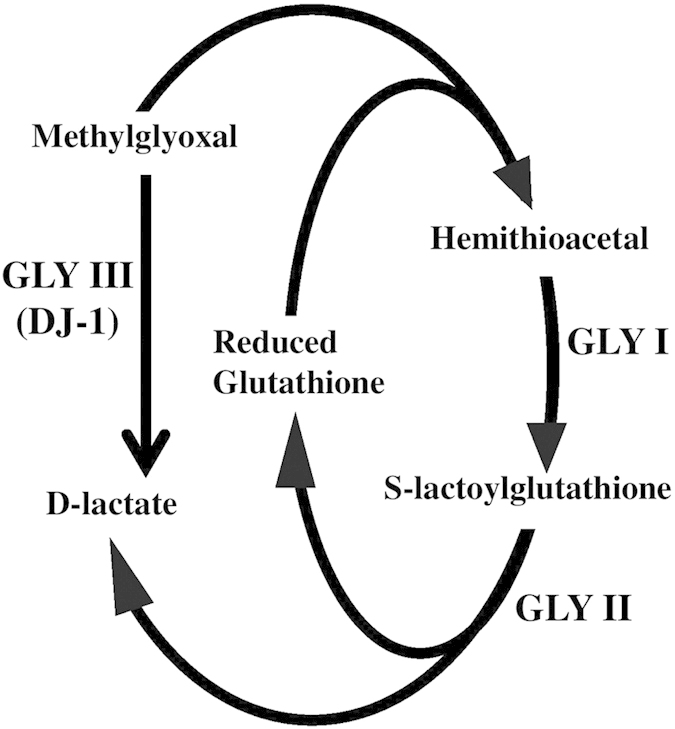
Unique glyoxalase pathway. Conventional glyoxalase pathway consists of two enzymes (GLY I and GLY II) that detoxify MG into D-lactate with the help of reduced glutathione. However, the novel glyoxalase enzyme (DJ-1/GLY III) is able to do the conversion in a single step without the help of any cofactor(s).

**Table 1:** Summary of DJ-1 proteins used in the study along with their source and accession numbers.

Sl. No.	Name of Species	Classification	Swiss-prot Ids	No. of members
1	*Oryza punctata*	Monocot	G9C2Z3	1
2	*Oryza officinalis*	Monocot	G9C3D7	1
3	*Aegilops tauschii*	Monocot	M8BGN0	1
4	*Oryza rufipogon*	Monocot	Q2L7J8	1
5	*Oryza minuta*	Monocot	G9C344, G9C378	2
6	*Brachypodium distachyon*	Monocot	I1GY83, I1HDA7, I1J382	3
7	*Triticum urartu*	Monocot	M7Y5 × 3, M7YUM7, M8AGC2	3
8	*Musa acuminata*	Monocot	M0RII5, M0RL94, M0SI74, M0TRZ6,	4
9	*Oryza sativa* subsp. *indica*	Monocot	A2XYU1, A2ZG23, B8AAK7, B8B006, B8B3B1	5
10	*Sorghum bicolor*	Monocot	C5XKH1, C5Y5C9, C5YA50, C5YMA2, C5Z3P7	5
11	*Oryza brachyantha*	Monocot	J3KXP0, J3M2I7, J3MEP9, J3MTR3, J3N9B7	5
12	*Setaria italica*	Monocot	K3XI28, K3XWS2, K3YHK0, K3Z6Y9, K3ZIS3	5
13	*Oryza glaberrima*	Monocot	I1NLA7, I1NLA9, I1PQS8, I1PXC5, I1Q2R6, I1R1B0	6
14	*Zea mays*	Monocot	B4F871, B4FPM1, B6TLL7, B6TU74, B6TVM7, C0PIH4, C0PIM0, K7VDN4, K7VH97, K7VUU0, K7UTZ8	11
15	*Oryza sativa* subsp. *japonica*	Monocot	Q0JPK7, Q5QNF2, Q5Z790, Q6F2Y9, Q7XPI5, B9FL63, B9FTL1, B9G8C7, A3BCD1, Q2R1V5, Q5QNE8, Q258Y7	12
16	*Triticum aestivum*	Monocot	W5AMX9, W5B873, W5BYM8, W5CKN3, W5CV43, W5DFB7, W5H8W7, W5HB49, W5HQR0, W5HZX7, W5I140, W5I1R5, W5IB79	13
17	*Hordeum vulgare* var. *distichum*	Monocot	F2CR89, F2CRA2, F2CUF4, F2CY81, F2D1P7, F2D2X9, F2DU80, F2DVS2, F2DW14, F2E8C5, F2EDR4, F2EG99, F2EGX7, M0V2D0,M0W2L7, M0WAD7,M0XZ96, M0YG85, M0YG87, M0YG88, M0YG89	21
18	*Thellungiella halophila*	Dicot	E4MXM5	1
19	*Arabidopsis halleri* subsp. *halleri*	Dicot	I0J3E1	1
20	*Capsicum chinense*	Dicot	B1Q484	1
21	*Lotus japonicus*	Dicot	I3SDB4	1
22	*Gossypium hirsutum*	Dicot	S4U1Y3	1
23	*Amborella trichopoda*	Dicot	W1PP51, W1Q086	2
24	*Genlisea aurea*	Dicot	S8CA72, S8CKY5, S8EM04	3
25	*Prunus persica*	Dicot	M5VM59, M5WGA9, M5WJY4	3
26	*Solanum lycopersicum*	Dicot	K4B323, K4BA10, K4CGU4	3
27	*Solanum tuberosum*	Dicot	M1AX34, M1BQT9, M1BQU0, M1CFB3	4
28	*Vitis vinifera*	Dicot	F6H2P8, F6I594, D7TA50, D7TPH6, A5AVI4	5
29	*Medicago truncatula*	Dicot	B7FLB0, G7J7V6, G7J7V8, G7J7W0, G7JL67	5
30	*Thellungiella salsuginea*	Dicot	V4L3Y1, V4M128, V4M4G1, V4M823, V4MFM5, V4MJ25	6
31	*Populus trichocarpa*	Dicot	U5FGI1, U7DVP5, B9H3J2, B9H3J4, B9HS15, A9PB55, B9I1V5	7
32	*Arabidopsis lyrata*	Dicot	D7KKG0, D7KZK5, D7L2Z4, D7L3X8, D7LCC6, D7LUV8, D7MFS6	7
33	*Phaseolus vulgaris*	Dicot	T2DMJ8, V5N8K4, V7BLU1, V7BTW5, V7CEB9, V7CHW7, V5N8C7	7
34	*Citrus clementina*	Dicot	V4S745, V4T235, V4T6C4, V4TQT3, V4TVP7, V4VRV0, V4VRW5	7
35	*Ricinus communis*	Dicot	B9RYH2, B9SE59, B9SJL8, B9SJL9, B9TFX6, B9TH23, B9TI86	7
36	*Capsella rubella*	Dicot	R0F261, R0FWX0, R0GWW2, R0H4F0, R0HM98, R0HZ27, R0I329	7
37	*Brassica rapa* subsp. *pekinensis*	Dicot	M4CBJ7, M4CG57, M4E9M5, M4EQ76, M4EXE3, M4FEZ4, M4FHL6, Q7XBC0, Q8H6J5	9
38	*Glycine max*	Dicot	C6TBE8, I1LV54, I1M322, I1MZJ8, K7K833, K7L305, K7L306, K7LRD9, K7MPX7, K7MPX8	10
39	*Arabidopsis thaliana*	Dicot	Q9FPF02, Q9FPF0, Q9M1G8, Q9M8R4, Q9MAH3, Q9ZV192, Q9ZV19, B3H6C6, Q56ZU8, Q56ZE0	10
40	*Physcomitrella patens* subsp. *patens*	Bryophyte	A9RPR5, A9T0G4	2
41	*Picea sitchensis*	Gymnosperm	A9P1Y3, D5AA19, D5AA85, D5AAF0	4
42	*Selaginella moellendorffii*	Lycopod	D8QW13, D8RRY6, D8SAX0, D8SJK8, D8SW04	5

**Table 2:** List of putative *DJ-1* genes in rice along with their chromosomal locations, alternative spliced forms, CDS, polypeptide length, isoelectric point, and localization etc (bp base pair, aa amino acid, Da daltons).

Gene	Protein	Chrom. No	Locus	CDS (bp)	CDS coordinate (5′ to 3′)	PP length (aa)	Mass (Da)	pI	Localization	Orthologous genes
*OsDJ-1A*	OsDJ-1A	1	LOC_Os01g11860.1	1168	6412786 – 6419895	396	40437.2	4.46	Cy^a^,^b^	GRMZM2G102811, GRMZM2G127812, Sb03g001750
*OsDJ-1B*	OsDJ-1B	1	LOC_Os01g11880.1	1287	6432336 – 6436086	429	45305.5	6.19	Cp^a^,^b^,^c^	Bradi2g07040, GRMZM2G024959, GRMZM2G102927, Sb03g001740
*OsDJ-1C*	OsDJ-1C.1	4	LOC_Os04g57590.1	1164	34270848 – 34267734	388	41329.3	5.22	Cy^a^,^b^	AT3G02720(AtDJ-1d), Bradi5g25750, GRMZM2G077541, GSVIVG00035277001, POPTR_0004s07440, POPTR_0004s07460, POPTR_0017s01530, POPTR_0017s01540, Sb06g032470
	OsDJ-1C.2		LOC_Os04g57590.2	1140	34270715 – 34267734	380	40407.1	5.37	Cy^a^,^b^	
	OsDJ-1C.3		LOC_Os04g57590.3	999	34269693 – 34267734	333	35535.6	5.04	Cy^a^, Cp^b^	
*OsDJ-1D*	OsDJ-1D.1	5	LOC_Os05g44330.1	1365	25796297 – 25803236	455	48673.6	8.57	Cp^a^,^b^,^c^, Mi^a^	AT4G34020(AtDJ-1c), Bradi3g38210, GRMZM2G117189, GSVIVG00031729001, POPTR_0009s10390, Sb07g022950
	OsDJ-1D.2		LOC_Os05g44330.2	1164	25796297 – 25803236	388	41532.2	8.29	Cp^a^,^b^,^c^	
	OsDJ-1D.3		LOC_Os05g44330.3	1147	25796297 – 25800798	349	37509.5	6.63	Cp^a^,^b^,^c^	
	OsDJ-1D.4		LOC_Os05g44330.4	717	25796297 – 25800798	239	25406.5	5.65	Cp^a^,^b^,^c^	
	OsDJ-1D.5		LOC_Os05g44330.5	1365	25796297 – 25803236	455	48673.6	8.57	Cp^a^,^b^,^c^, Mt^a^	
*OsDJ-1E*	OsDJ-1E	6	LOC_Os06g3404 0.1	1332	19829657 – 19824521	444	46877.6	7.74	Cp^a^,^b^,^c^	AT1G53280(AtDJ-1b), AT3G14990(AtDJ-1a), Bradi1g38760, GRMZM2G072909, GSVIVG00014220001, POPTR_0011s11320, Sb10g020910
*OsDJ-1F*	OsDJ-1F	11	LOC_Os11g3792 0.1	1191	22491418 – 22493083	397	41007.1	6.05	Cp^a^,^b^, Cy^a^, PM^a^	AT2G38860(AtDJ-1e), AT3G54600 (AtDJ-1f), Sb05g022900

Abbreviations: Cp chloroplast, Cy cytosol, Mt mitochondrial, PM plasma membrane, CDS coding DNA sequence.

^a^Localization prediction by CELLO v.2.5 (http://cello.life.nctu.edu.tw/)

^b^Localization prediction by pSORT (http://wolfpsort.org/ )

^c^Chloroplast localization signal confirmed by ChloroP (http://www.cbs.dtu.dk/services/ChloroP/).

**Table 3:** Comparison of kinetic parameters of reported GLY III enzymes from various organisms.

Organism	Protein	*K*_m_(mM)	*k*_cat_ (min^−1^)	*k*_cat_/*K*_m_(min^−1^M^−1^)	Reference
Rice	OsDJ-1C	0.74	2500	3.36 × 10^6^	Present study
Bacteria	EcGLY III	1.43	156.9	1.1 × 10^5^	15
Human	HsDJ-1	0.60	72.38	1.21 × 10^5^	18
Arabidopsis	AtDJ-1a	5.48	102	1.9 × 10^4^	33
Arabidopsis	AtDJ-1b	4.16	154	3.7 × 10^4^	33
Arabidopsis	AtDJ-1d	0.1	1700	17 × 10^6^	33
Fission yeast	SpDJ-1	10.8	85.7	7.9 × 10^3^	48
Fungus	CaGlx3	5.5	468	8.5 × 10^4^	29
