# Supplementary material for: Presence of unique glyoxalase III proteins in plants indicates the existence of shorter route for methylglyoxal detoxification
Source: Sci Rep. 2016 Jan 6;6:18358. doi: 10.1038/srep18358 (PMC4702089; doi:10.1038/srep18358)
Supplement: Supplementary Information [file srep18358-s1.pdf]

Presence of unique glyoxalase III proteins in plants indicates the existence of shorter route for methylglyoxal detoxification

Ajit Ghosh[1,3], Hemant R Kushwaha[1], Mohammad R Hasan[1], Ashwani Pareek[2], Sudhir K Sopory[1], Sneh L Singla-Pareek[1,*]

[1]Plant Molecular Biology, International Centre for Genetic Engineering and Biotechnology, Aruna Asaf Ali Marg, New Delhi-110067, India, [2]Stress Physiology and Molecular Biology Laboratory, School of Life Sciences, Jawaharlal Nehru University, New Delhi-110067, India, and [3]Present address: Department of Biochemistry and Molecular Biology, Shahjalal University of Science and Technology, Sylhet-3114, Bangladesh.

*To whom correspondence should be addressed:

Sneh L Singla-Pareek, Plant Molecular Biology Group, International Centre for Genetic Engineering and Biotechnology, Aruna Asaf Ali Marg, New Delhi-110067, India.

Phone: +91-11-26741358, Ext. 363 (O);

Fax: +91-11-26742316;

E-mail: sneh@icgeb.res.in

Table S1| Comparison of enzyme kinetic parameters of WT OsDJ-1C protein and its mutein C119A

Sl. no	Parameter	WT	C119A	Deviation from WT (%)
1	K_m (μM)	744	602	19
2	V_{max} (μmol $min^{-1}mg^{-1}$)	58	22	62
3	k_{cat} (min^{-1})	2500	945	62
4	k_{cat}/K_m (mM^{-1} min^{-1})	3359	1569	53

Table S2 | List of single domain containing DJ-1 proteins in plants used in this study

Sl. No.	Name of Species	Classification	Swiss-prot Ids
1	*Oryza sativa subsp. japonica*	Monocot	A3BCD1, B9G8C7
2	*Hordeum vulgare var. distichum*	Monocot	F2D1P7, F2EG99, M0V2D0, M0WAD7, M0XZ96, M0YG87
3	*Triticum urartu*	Monocot	M7Y5X3
4	*Triticum aestivum*	Monocot	W5CKN3, W5CV43, W5DFB7, W5H8W7
5	*Zea mays*	Monocot	K7VDN4, K7VH97
6	*Sorghum bicolor*	Monocot	C5YMA2
7	*Arabidopsis thaliana*	Dicot	B3H6C6, Q56ZE0, Q56ZU8
8	*Medicago truncatula*	Dicot	B7FLB0
9	*Ricinus communis*	Dicot	B9TFX6
10	*Vitis vinifera*	Dicot	F6I594
11	*Glycine max*	Dicot	K7K833
12	*Brassica rapa subsp. pekinensis*	Dicot	Q8H6J5
13	*Phaseolus vulgaris*	Dicot	V7CEB9
14	*Populus trichocarpa*	Dicot	U7DVP5
15	*Genlisea aurea*	Angiosperms (Eudicots)	S8CKY5

Table S3 | List of *DJ-1* genes in *Arabidopsis*, along with their chromosomal locations, alternative spliced forms, CDS, polypeptide length, molecular weight, and localization (bp base pair, aa amino acid, Da daltons)

**Swiss-prot ID**	**Gene**	**Protein**	**Chromosome no.**	**Locus**	**CDS (bp)**	**Polypeptide length (aa)**	**Mol. Mass (Da)**	**pI**	**Localization**
Q9MAH3	*AtDJ-1b*	AtDJ-1b	1	AT1G53280	1317	438	46990.1	7.95	Chloroplast
Q9ZV19	*AtDJ-1e*	AtDJ-1e.1	2	AT2G38860.1	1170	389	41668.1	5.01	Cytoplasm
		AtDJ-1e.2		AT2G38860.2	1197	398	42729.2	4.84	
		AtDJ-1e.3		AT2G38860.3	840	279	30133.6	4.37	
Q9M8R4	*AtDJ-1d*	AtDJ-1d	3	AT3G02720	1167	388	41645.3	5.08	Cytoplasm
Q9FPF0	*AtDJ-1a*	AtDJ-1a.1	3	AT3G14990.1	1179	392	41856.9	5.08	Vacuole, Plasma
		AtDJ-1a.2		AT3G14990.2	1110	369	39478.1	5.07	membrane,Chloroplast,
		AtDJ-1a.3		AT3G14990.3	1110	369	39478.1	5.07	Plasmodesmata, Cytosol
Q9MIG8	*AtDJ-1f*	AtDJ-1f	3	AT3G54600	1200	399	43141.7	5.65	Cytosol
Q56ZU8	*AtDJ-1c*	AtDJ-1c.1	4	AT4G34020.1	1419	472	50986.1	8.99	Chloroplast
		AtDJ-1c.2		AT4G34020.2	1314	437	47336.0	9.07	

Table S4 | Comparison of enzyme kinetics of conventional GLY I/II enzymes with the novel GLY III enzymes from various species

Species	Protein	K_m (μM)	k_{cat} (s^{-1})	k_{cat}/K_m ($M^{-1}s^{-1}$)	Reference
Oryza sativa	OsGLYI-11.2	100	70.96	$7.1X10^5$	1
	OsGLYII-2	254	508	$2.0X10^6$	2
	OsDJ-1C	744	41.66	$5.6X10^4$	Present study
E. coli	EcGLYI	27	338	$1.2X10^7$	3
	EcGLYII	184	53	$4.7X10^5$	4
	EcGLY III	1430	2.62	$0.1X10^4$	5
Homo sapiens	HsGLYI	66	500	$2.3X10^7$	6
	HsGLYII	187	780	$4.2X10^6$	7
	HsDJ-1	600	1.21	$0.2X10^4$	8

Table S5| List of primer pairs and their sequence used for qRT-PCR analysis and site directed mutagenesis

Sl. no	Gene name	Primer name	Sequence (5'-3')	Product size
1	*OsDJ-1A*	DJ-1A_for1	ATGCAGATGTGTGATCTGTGA	147
		DJ-1A_rev1	TCCATCAATCAATCCATCAGCTA	
2	*OsDJ-1B*	DJ-1B_for2	GCCTGTCACCTGTGAGTGAT	177
		DJ-1B_rev2	AGACAGCGTAAACTCACCGA	
3	*OsDJ-1C*	DJ-1C_for1	CACAGCTTTGGGCTTTCGTC	147
		DJ-1C_rev1	CAGCTCCTTCTGCTCGTACA	
4	*OsDJ-1D*	DJ-1D_for1	GCGGTTGAGAGACAGTGTGA	108
		DJ-1D_rev1	CACGGCATGAGAACAACAGC	
5	*OsDJ-1E*	DJ-1E_for1	TGAGGCATCCTTTTGCTCGT	147
		DJ-1E_rev1	ACCCAAAGGTGCAATATCCATC	
6	*OsDJ-1F*	DJ-1F_for1	CTCGCCGAGTTCATCGCTC	99
		DJ-1F_rev2	TGACAGAGAGAACAGAGAAGCA	
7	*eEF-1α*	eEF_for	TTTCACTCTTGGTGTGAAGCAGAT	100
		eEF_rev	GACTTCCTTCACGATTTCATCGTAA	

References:

1. Mustafiz, A. *et al.* A unique Ni -dependent and methylglyoxal-inducible rice glyoxalase I possesses a single active site and functions in abiotic stress response. *Plant J.* **78**, 951-963 (2014).
2. Ghosh, A., Pareek, A., Sopory, S. K. & Singla-Pareek, S. L. A glutathione responsive rice glyoxalase II, OsGLYII-2, functions in salinity adaptation by maintaining better photosynthesis efficiency and anti-oxidant pool. *Plant J.* **80,** 93-105 (2014).
3. Clugston, S. L. *et al*. Overproduction and characterization of a dimeric non-zinc glyoxalase I from *Escherichia coli*: evidence for optimal activation by nickel ions. *Biochemistry* **37,** 8754-8763 (1998).
4. O'Young, J., Sukdeo, N. & Honek, J. *Escherichia coli* glyoxalase II is a binuclear zinc-dependent metalloenzyme. *Arch. Biochem. Biophys.* **459,** 20-26 (2007).
5. Subedi, K. P., Choi, D., Kim, I., Min, B. & Park, C. Hsp31 of *Escherichia coli* K-12 is glyoxalase III. *Mol. Microbiol.* **81,** 926-936 (2011).

6. Ridderstrom, M., Cameron, A. D., Jones, T. A. & Mannervik, B. Mutagenesis of residue 157 in the active site of human glyoxalase I. *Biochem. J.* **328,** 231-235 (1997).
7. Ridderstrom, M. *et al*. Molecular cloning, heterologous expression, and characterization of human glyoxalase II. *J. Biol. Chem*. **271,** 319-323 (1996).
8. Lee, J. Y. *et al.* Human DJ-1 and its homologs are novel glyoxalases. *Hum. Mol. Genet.* **21**, 3215-3225 (2012).

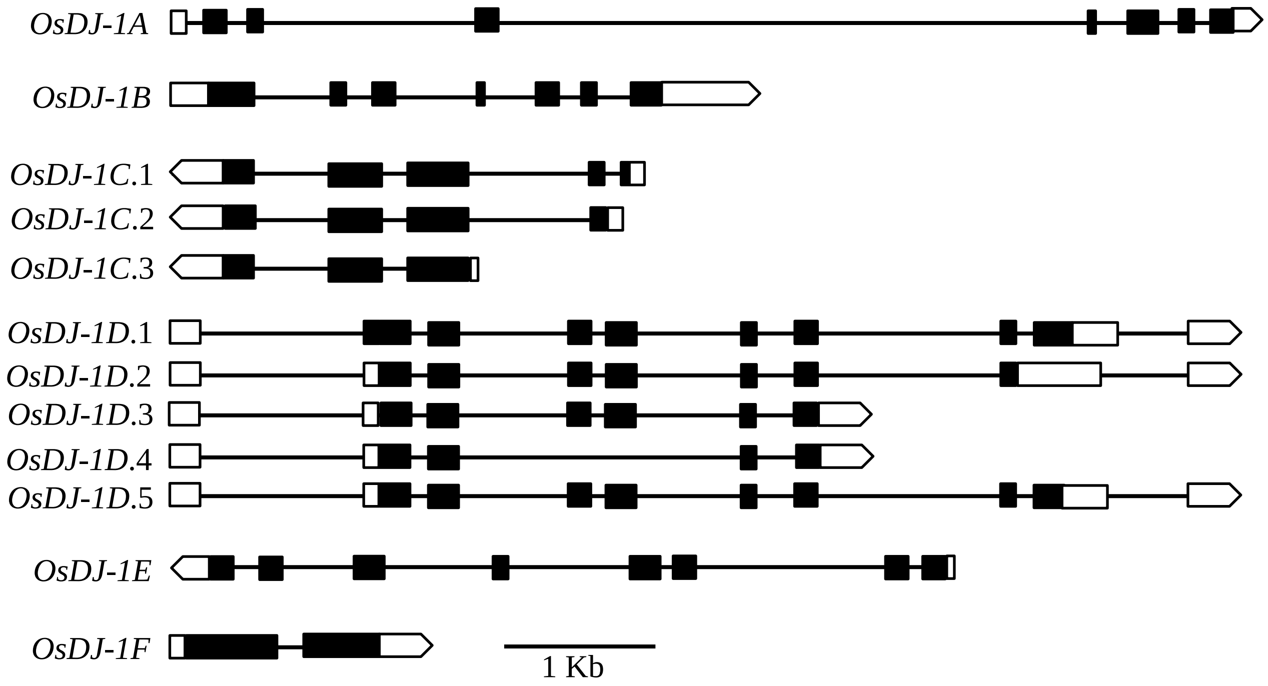

Figure S1| Gene structures of all the *DJ-1* family members of rice including alternative spliced forms. All the exons are shown in filled black boxes and the introns are indicated by black lines. The 5'-UTR regions are shown using empty boxes and the 3'-UTR regions are shown in empty arrows that indicates the direction of the gene.

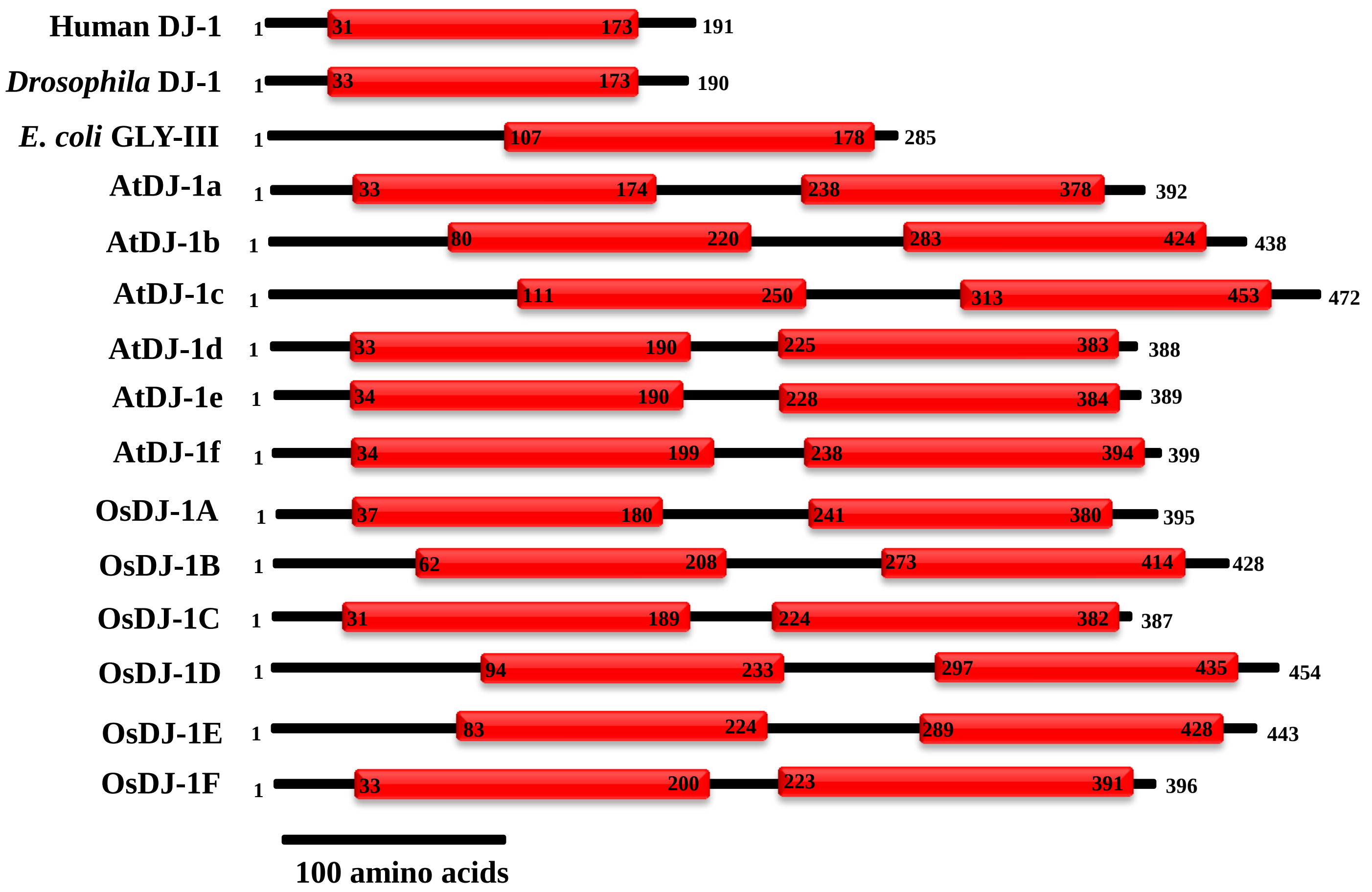

Figure S2| Domain architecture of various GLYIII/DJ-1 proteins. All the *Arabidopsis* and rice DJ-1 proteins along with human, *Drosophila* and *E. coli* GLY-III proteins were analyzed for the presence of DJ-1 domain. All plant DJ-1 proteins possess two DJ-1 domains (represented by red box), whereas non-plant proteins possess only one DJ-1 domain. The length of full protein as well as domain(s) are indicated by exact amino acid numbers.

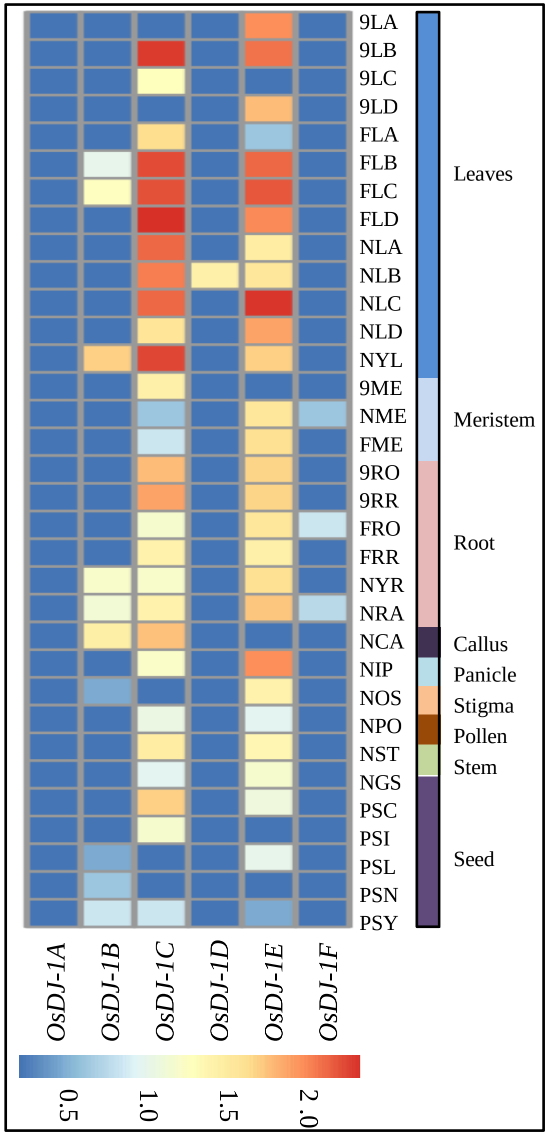

Figure S3| Expression profiling of *OsDJ-1* genes using MPSS data in different tissues of rice. Heatmap has been generated using TIGR MeV software package and represent hierarchical clustering of average log_2 signal values. The color bar below represents relative expression values; thereby, red color represents highest expression levels, white represents medium expression levels, and blue signifies lowest expression levels.

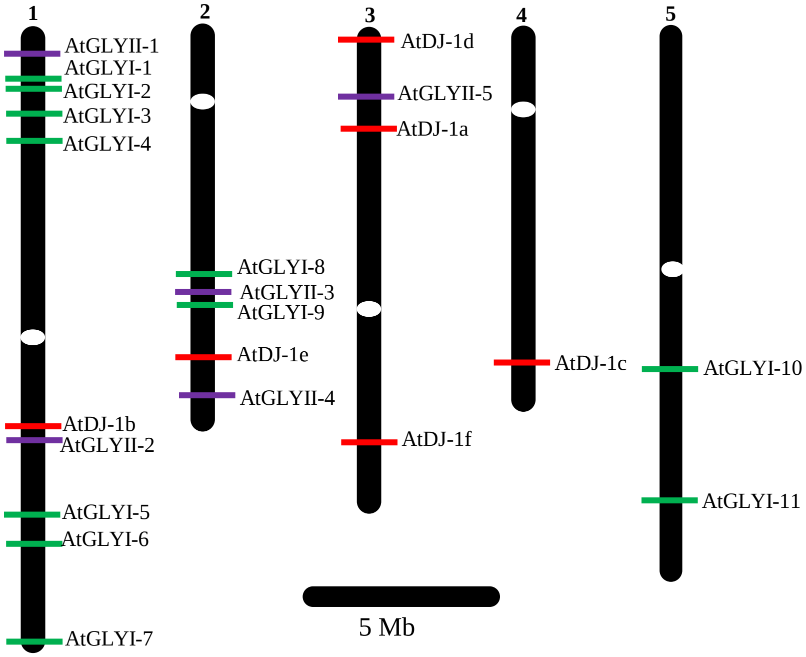

Figure S4| Chromosomal distribution of glyoxalase genes (GLYI, GLYII and DJ-1) in *Arabidopsis*. Chromosomes carrying glyoxalase genes are shown with their respective numbers at the top of the bar. Novel glyoxalase III (DJ-1) genes have been marked along with GLY I and GLY II genes. GLY I, GLY II and DJ-1 genes are marked with green, purple, and red colour boxes respectively.

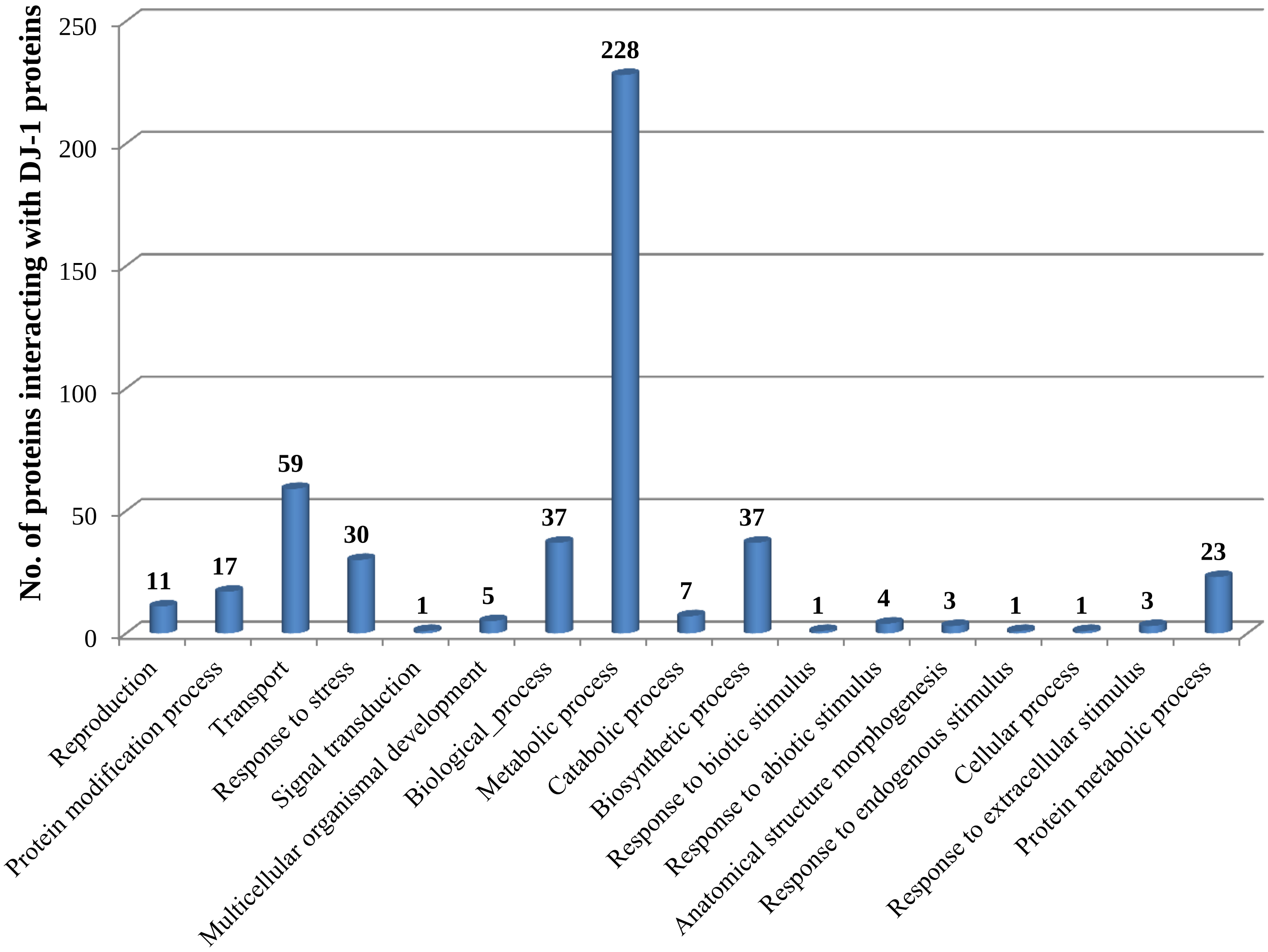

Figure S5| *In silico* prediction of proteins interacting with OsDJ-1 proteins in rice.
